# Evaluating Drug Effectiveness for Antihypertensives in Heart Failure Prognosis: Leveraging Composite Clinical Endpoints and Biomarkers from Electronic Health Records

**DOI:** 10.1145/3584371.3612977

**Published:** 2023-10-04

**Authors:** Shaika Chowdhury, Yongbin Chen, Xiao Ma, Qiying Dai, Yue Yu, Nansu Zong

**Affiliations:** Department of Artificial Intelligence and Informatics Research, Mayo Clinic, Rochester, MN, USA; Department of Biochemistry and Molecular Biology, Mayo Clinic, Rochester, MN, USA; Department of Cardiovascular Medicine Mayo Clinic, Rochester, MN, USA; Department of Cardiovascular Medicine, Mayo Clinic, Rochester, MN, USA; Department of Health Sciences Research, Mayo Clinic, Rochester, MN, USA; Department of Artificial Intelligence and Informatics Research, Mayo Clinic, Rochester, MN, USA

**Keywords:** Electronic health records, Drug effectiveness, Heart failure, Annotation, Deep learning

## Abstract

Arterial hypertension is a major risk factor for heart failure and antihypertensives such as angiotensin converting enzyme (ACE) inhibitors and β-blockers are considered as its first-line treatment. Drug response prediction models designed to determine the most effective antihypertensive drug for a patient are hindered by the interpatient response variability. Although typically pharmacogenetic data have been used to investigate the association of genetic variants with the antihypertensive response, genomewide association studies are currently expensive and the translation of genotype guided antihypertensive therapy to clinical practice is challenging. With the generation of electronic health records (EHR) data summarized over the patient’s disease prognosis and interventions, it is still an underused resource for antihypertensive effectiveness studies in heart failure management. In this study, we first use the clinical events in the EHR related to the patient’s hard clinical endpoints and biomarkers associated with the heart failure condition to design selection strategies that determine the antihypertensive effectiveness, then develop annotated corpora using the strategies and eventually evaluate supervised deep learning classifiers on the annotated data. We annotated the EHR sequences of approximately 9500 patients with binary labels corresponding to the drug effectiveness across two different antihypertensive classes and our trained classifier was able to obtain the best F1 performance of 0.97.

## 1 Introduction

With a prevalence of > 37.7 million globally [1], heart failure (HF) remains as the only cardiovascular disease that is characterized by an increased rate of overall mortality albeit progress in its diagnosis and management [2]. Statistically, it is estimated that 35% of HF patients pass away within the first year [3], while 1 in 4 HF patients are readmitted within 30 days of discharge [4]. An elevation of blood pressure (BP), known as hypertension, represents the leading modifiable risk factor in the development of HF and is responsible for approximately 9.4 million deaths per year [5]. The introduction of antihypertensive treatment has shown to reduce the risk of heart failure through BP lowering [6]. In particular, compared to placebo in clinical trials, angiotensin converting enzyme inhibitors (ACEI) contributed to a reduction in HF by 18% and β-blockers (BB) displayed similar effects [7]. However, interpatient variability has been observed in the antihypertensive drug response with patients responding differently to the same drug [8]. This results in a lack of drug efficacy in some patients as it has been found that 50% of patients fail to respond to antihypertensive monotherapy [9]. Hence, alternative approaches are required to identify the most appropriate antihypertensive therapy for individual patients.

Recent years have seen a surge in the development of computational drug response models [10] for the purpose of individualizing the selection of drugs to optimize patient treatment. An important determinant in the performance of these computational models is the annotated data used for the model training and evaluation to guide the drug decision-making. Although there is an uptake of pharmacogenetic data in the antihypertensive drug-response studies due to polymorphisms in genes, the associations of these polymorphisms with the modified drug response is small and irreplicable leading to limited clinical implications [11, 12].

Previous research on clinical trials for HF treatment recommends the use of a composition of hard clinical endpoints as objectively evaluating the drug efficacy through the detection of adverse events (e.g., mortality, hospitalization) [13] to accurately reflect the patient’s HF status. While biomarkers, namely, B-type natriuretic peptide (BNP) and N-terminal pro-B-type natriuretic peptide (NT-proBNP) are physiological measures predictive of HF progression, determined on the basis of their percentage change in concentration [14], and are deemed as surrogate endpoints in monitoring HF treatment response [15]. With the accumulation of observations related to the patient’s medical history and laboratory tests in the Electronic health records (EHR), it is possible to extract information related to the clinical endpoints and biomarkers with potential to guide the drug effectiveness annotation; yet EHR remains an underused data source in drug effectiveness studies despite being more accessible.

In this study, we develop annotated corpora for antihypertensive effectiveness prediction of HF patients via two rule-based approaches, with rules derived from the EHR data based on the clinical endpoints and biomarkers associated with HF. We then train deep learning classifiers on the patients’ annotated EHR sequences to automatically determine through binary classification if the antihypertensive treatment across two different drug classes (i.e., ACEI, BB) is effective. The corpora currently consist of 2357 positive patients (i.e., *antihypertensive is effective*) and 2430 negative patients (i.e., *antihypertensive is not effective*) with respect to ACEI evaluation and 3459 positive patients and 3763 negative patients with respect to BB evaluation.

## 2 EHR Data

We use the patient information from the United Data Platform (UDP), the clinical data repository of Mayo Clinic. It is an exhaustive clinical data warehouse that contains millions of patients’ data, which are also updated in real time. It provides a combined view of multiple data sources collected from various clinical and hospital systems within the Mayo Clinic. In this study, we use the mortality, hospitalizations and lab tests information recorded as longitudinal clinical events over the patients’ HF-related encounters in the UDP’s EHR database. Included lab tests are BNP, NT-proBNP, diastolic blood pressure (DBP) and systolic blood pressure (SBP).

## 3 Annotation Pipeline

Our annotation pipeline illustrated in Figure 2 includes three steps: (1) Data filtering (2) Rule-based annotation schemes (3) Training and evaluation with supervised deep learning classifiers.

### 3.1 Data Filtering

**Figure 1:**
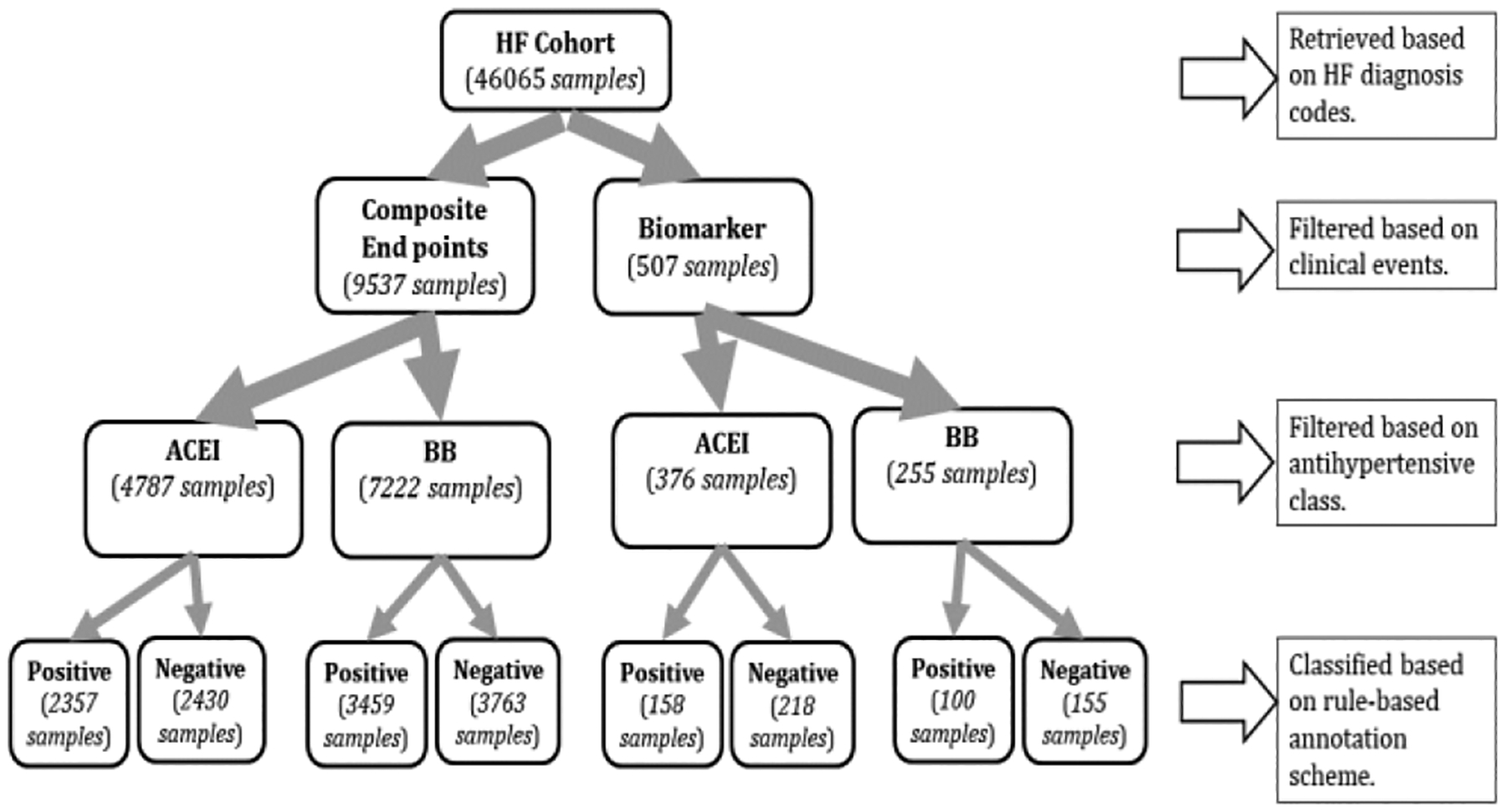
Data Filtering Workflow. Data size (number of patients) linked to each cohort based on the filtering step is shown within parentheses.

**Figure 2:**
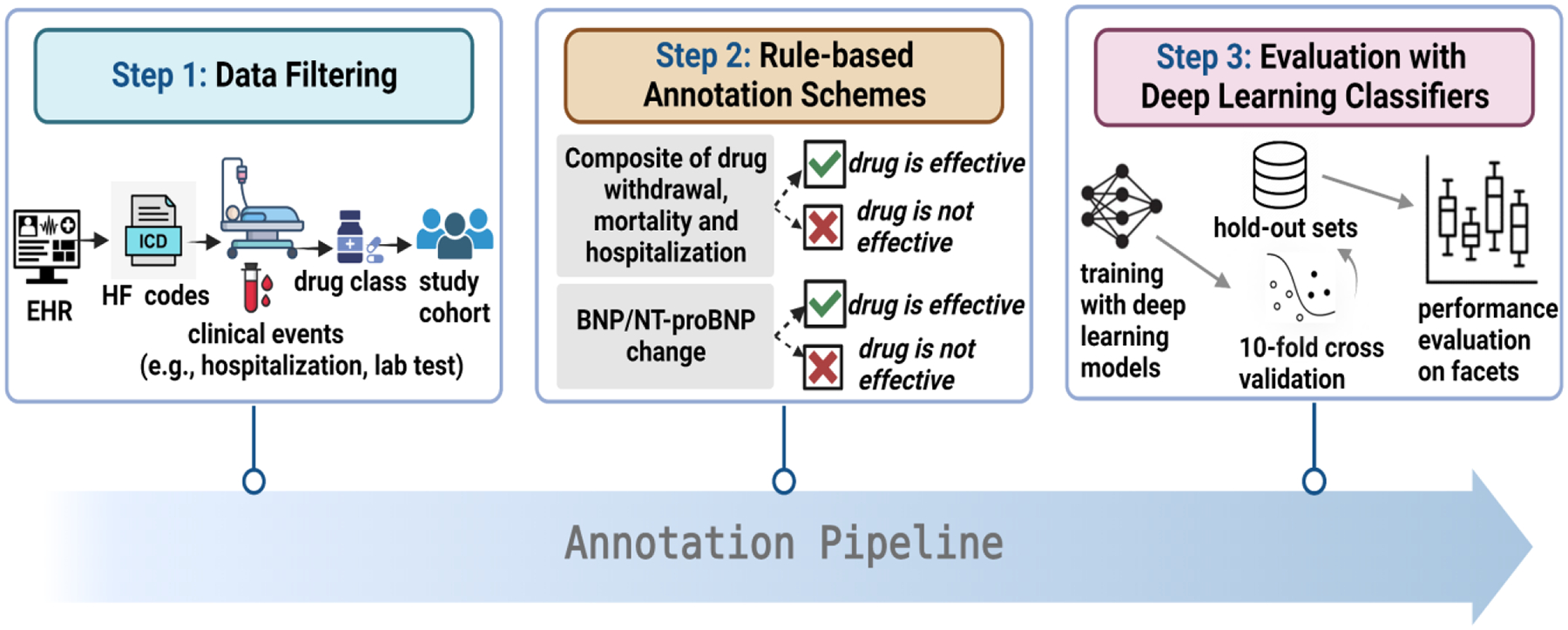
Annotation pipeline for drug effectiveness using EHR.

Our data filtering workflow is shown in Figure 1. Initially, we identify the patients using diagnosis codes associated with HF. We then filter based on the clinical events relevant to the two annotation schemes discussed in Section 3.2. For the composite endpoint-based scheme, we check the availability of clinical events related to the mortality and at least two HF-related hospitalizations. While for the biomarker-based scheme, we check the availability of BNP or NT-proBNP lab assessments over at least two HF-related hospitalizations. We also ensure a percentage decrease in the biomarker concentration within 25–50%. We then filter based on the antihypertensive class to ensure that the patient was taking a medication from either ACEI or BB. For the patients remaining after this step, we run the respective rule-based annotation scheme (next section) to classify patients into two types: (i) a positive label means that the antihypertensive treatment is effective; (ii) a negative label means that antihypertensive is not effective.

### 3.2 Rule-based Annotation Schemes

We design two different annotation schemes based on the composite of hard clinical outcomes and the change in biomarker level, respectively, observed over the patient’s HF prognosis. We had attempted to combine the two schemes into one but that resulted in data sparsity.

#### 3.2.1 Composite Endpoints

As revealed by a seminal work on drug efficacy for clinical trials [13], two types of endpoints are considered clinically meaningful in the evaluation of drugs for HF treatment. The first type evaluates any changes in the patient’s clinical status, while the second evaluates the occurrence of any major clinical event (e.g., death, hospitalization). For the former, New York Heart Association (NYHA) functional classification is used to assess the functional capacity of HF patients based on the severity of symptoms and is considered to provide subjective evidence. Whereas the latter is viewed as a more objective measure as death and hospitalization define definitive changes in the HF progression, thus being less susceptible to observer bias.

Motivated by this work, we design a composite selection strategy centered on the second type of measure to separate the positive patients from the negative patients as EHRs also encompass information related to morbidity and mortality. We also wanted to incorporate the first type of evaluation in our selection criteria, however, based on our early data processing we found that almost all the samples in our cohort belonged to the same NYHA class (i.e., 2–3), which was not distinctive enough for binary class division.

**Figure 3:**
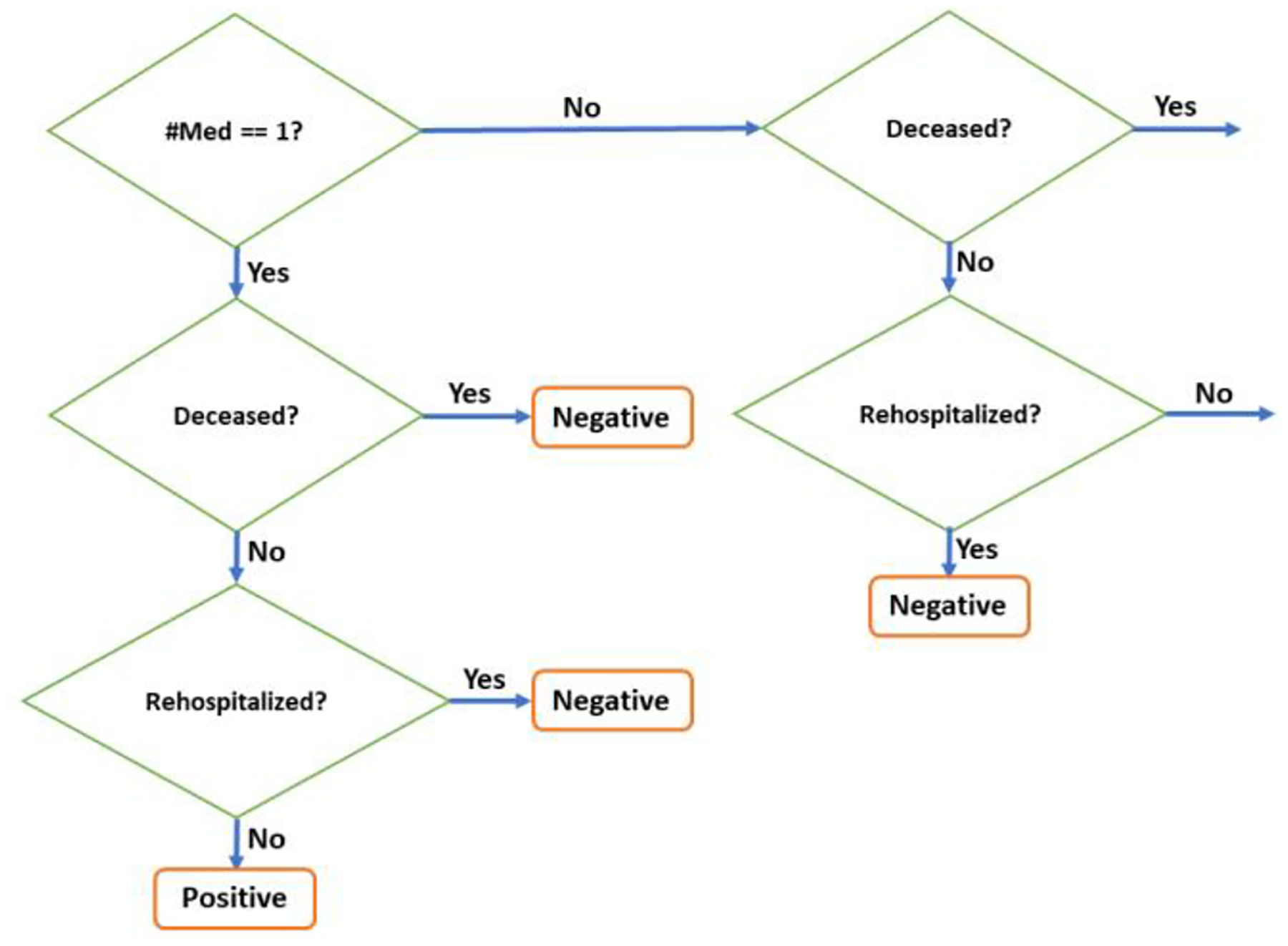
Flowchart depicting the composite selection strategy.

The proposed composite selection strategy is composed of three clinical measures structured as cascaded conditions for more clinically meaningful positive and negative samples, as shown in Figure 3. First, we check for drug withdrawal per antihypertensive class. This is based on the intuition that if the total number of medications for that class in the patient’s encounter history is one, then that could mean that the initially prescribed drug was never replaced with a different drug and hence is a possible indication of drug effectiveness. Nonetheless, reasons for drug withdrawal could also be influenced by the physician’s judgment or other administrative factors [19]. So, we check against two objective end points – mortality and rehospitalization - for further validation. If both are false (“No”), then that means the patient did not pass away during the HF treatment route and was not hospitalized for any worsening symptoms, affirming that the drug was effective on the patient. This forms our positive samples. Otherwise, if either of the measures is true (“Yes”) then we assign it as a negative sample. Note that to only count the significant hospitalizations as endpoints, we verify that the minimum duration is 24 hours and the time difference between the current encounter’s admit time and the previous encounter’s discharge time <= 24 hours. Refer to the first condition again for false branching of the number of medications. In this case, there are multiple medications involved in the patient’s encounters so we cannot directly attribute the cause of mortality or rehospitalization to the first medication. To mitigate these effects, we only consider the encounters associated with the first medication and analyze the truncated encounter sequence for mortality and rehospitalization. Subsequently, the patient being alive but hospitalized could be traced to the drug not being effective, forming a negative sample.

#### 3.2.2 Biomarker Change

Prognostic studies have shown that the assessment of the BNP/NT-proBNP concentration after treatment is predictive of cardiovascular events [16]. In particular, a percentage decrease in the concentration between 25–50% could be indicative of therapeutic effectiveness. Based on this empirical finding, we annotate patients as positive if the BNP/NT-proBNP measurement assessed in the last encounter decreases compared to that in the first encounter and the percentage decrease is significant (25–50%).

### 3.3 Training and Evaluation with Supervised Deep Learning Classifiers

We train and test supervised deep learning models on the two annotated datasets separately. We use the DBP and SBP lab observations as the features during training.

All experiments are executed using 10-fold cross-validation (CV) to robustly evaluate the performance of each model. We quantify the evaluation using several metrics - Accuracy, AUC-ROC, AUPRC, Precision, Recall and F-1 score. Furthermore, we stratified each fold to ensure the same proportion of positive and negative samples. Each model is trained for 100 epochs in a batch size of 32 using Adam [37] as the optimizer. Implementations of all models are done in TensorFlow v2 [38]. The code is available https://github.com/bioIKEA/HF_response_classification.

We experiment with the following deep learning models: *Multi-layer Perceptron (MLP*) with one hidden layer of 50 hidden units, *LSTM* [17] with 50 hidden units, *Stacked LSTM (S-LSTM)* composed of 2 LSTM layers stacked together, *Bi-LSTM* [18], which consists of 2 LSTM layers processing the input in opposite directions to facilitate capturing both the previous and future contexts, *CNN-LSTM* [19] and *Transformer* [20], which is a non-sequential model and uses self-attention mechanism to process the input as a whole allowing parallel computation. We set the number of attention heads to 1 as a higher number of heads performed worse in our hyperparameter-tuning experiments. The CNN-LSTM is a hybrid network that first consists of a convolutional neural network (CNN) component to capture the local information in the input using a 1D convolution and a 1D max-pooling layers. The output is then passed through an LSTM component for the modeling of temporal information in the input. We set the kernel size to 1 and the number of filters to 64 in the CNN-LSTM. We use rectified linear unit (ReLU) [21] as the activation function in all models. Hyperparameter-tuning is done on an independent validation set using grid search over a range of values as shown in Table 1.

## 4. Results

We evaluate the deep learning models on three facets – *annotation scheme*, *feature* and *antihypertensive class* – for fine-grained analysis. To inspect the contribution of each facet and find the facet combination deriving the best performance, we separately visualize box plots for each facet. When generating the box plots with respect to a facet, the values in relation to the facet form the x-axis, while the other two facets are set to fixed values so as to emphasize the effect that the values of the current facet have on the drug effectiveness performance in a comparative manner. Each box plot shows the distribution of the particular neural network’s performance on the 10-fold test data associated with the facet. We also perform a Student’s t-test to highlight the difference in the performance between the corresponding models through computation of the p-value (ρ). In each figure, we annotate the p-values with placeholder annotations denoting the range the p-value lies in to mark the degree of statistical significance. The annotation ‘ns’ stands for not statistically significant difference and star/s indicate statistical significance, such that the higher the number of stars the more statistically significant the difference.

**Figure 4:**
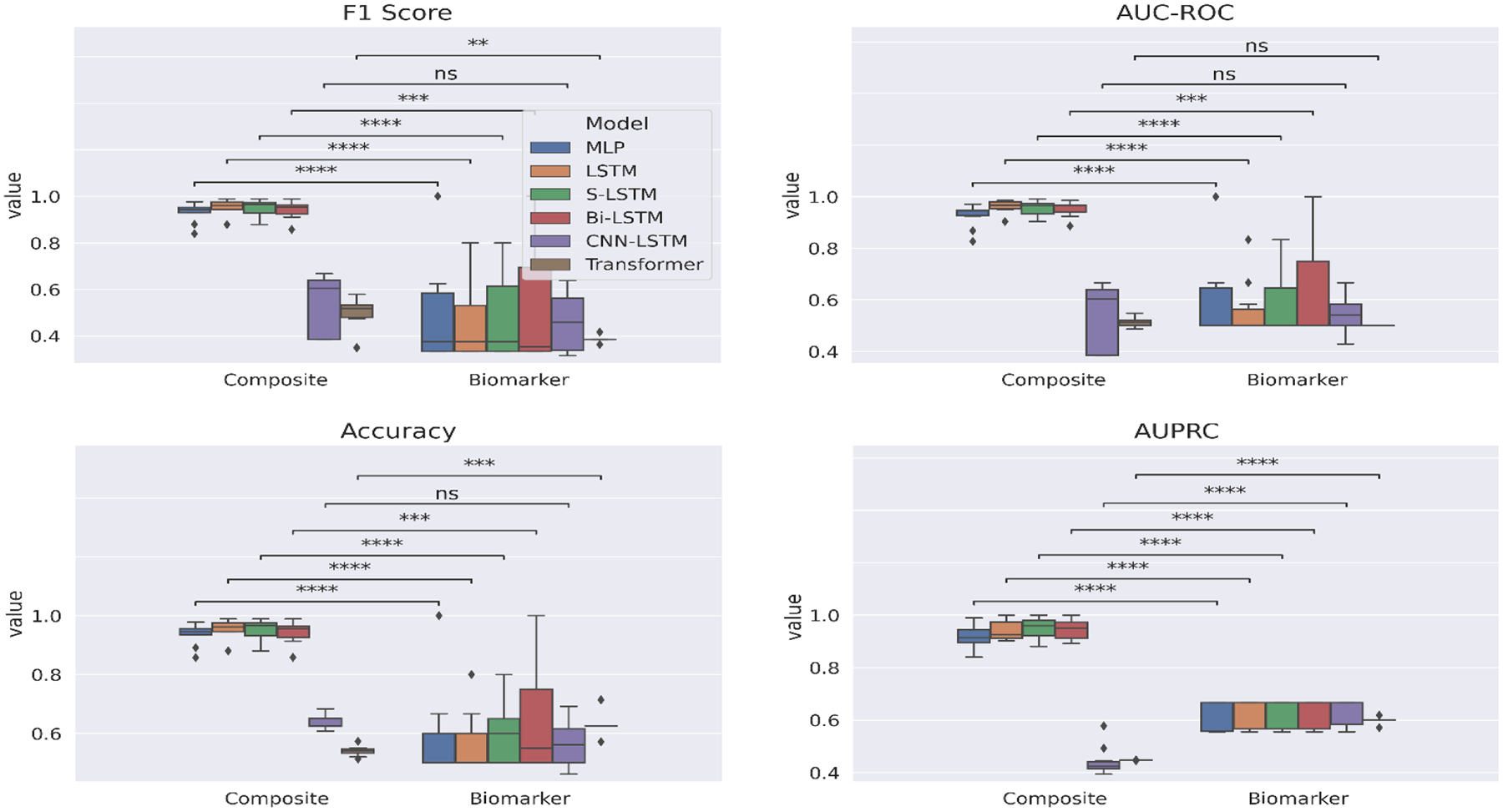
10-fold cross-validation evaluation of deep learning models on the facet *annotation scheme*.

Figure 4 reports the comparison in performance of the deep learning models on the facet annotation scheme. We hardcode the remaining two facets, feature and antihypertensive class, to the values DBP and ACEI respectively. Thus, this evaluation is carried out to assess the discriminative quality of the annotations in successfully predicting the effectiveness of ACEI therapy. The composite endpoint-based corpus is seen to generally perform better with statistical significance (p < 0.05) compared to the biomarker-based annotations. The best performing deep learning model is LSTM and its variant S-LSTM has comparable performance. A possible reason for the biomarker-guided annotations performing much worse could be the heterogeneous pathophysiology of HF that lead to variability in the physiological measurements across patients.

**Figure 5:**
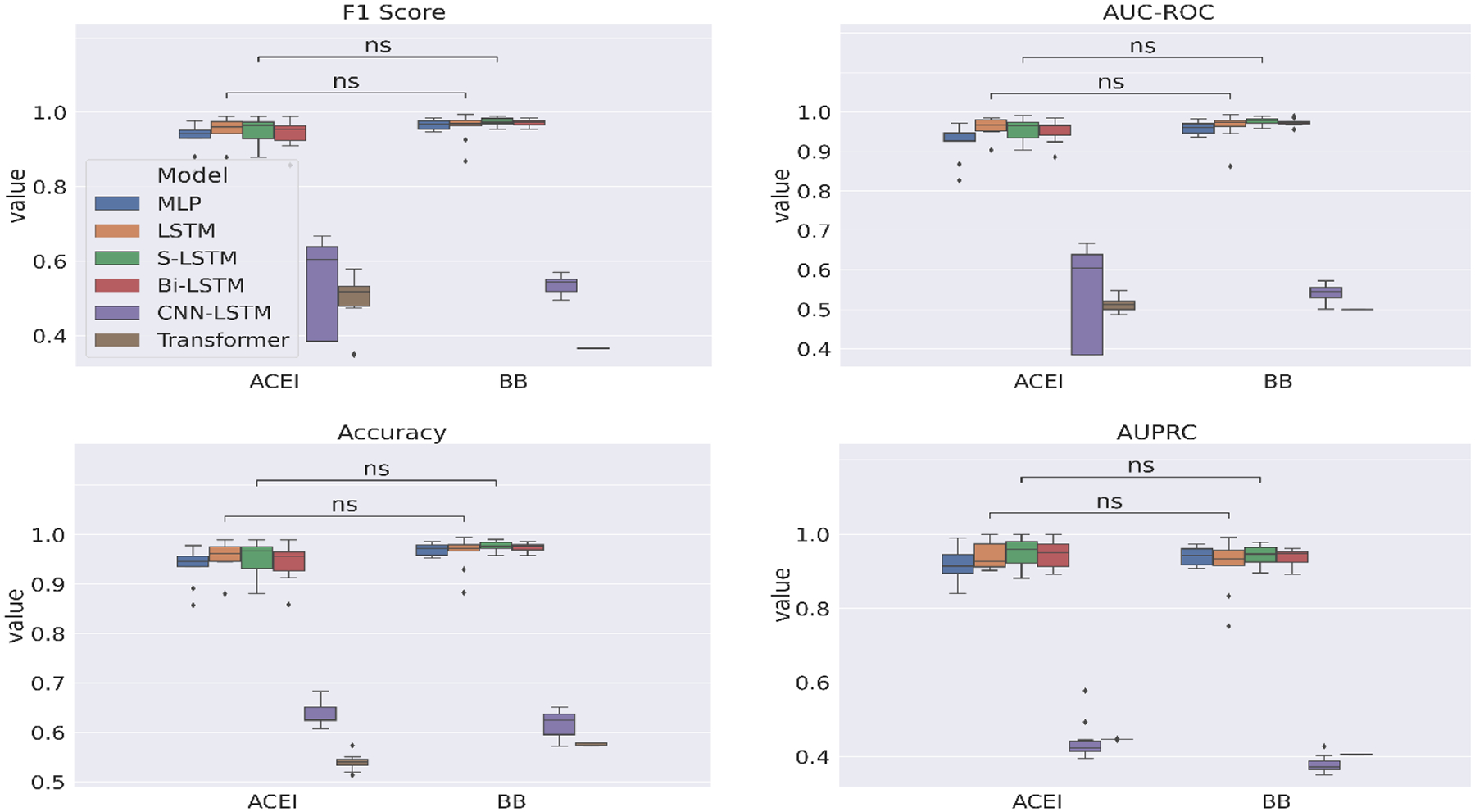
10-fold CV evaluation of deep learning models on the facet *antihypertensive class*.

Figure 5 plots the experimental results with the facet antihypertensive class which includes ACEI and BB. We allocate the remaining facets the best performing values based on the previous evaluation, namely Composite and DBP, and show the statistical significance for only the best performing models LSTM and S-LSTM. The differences in performances across the antihypertensives are overall not statistically significant.

**Figure 6:**
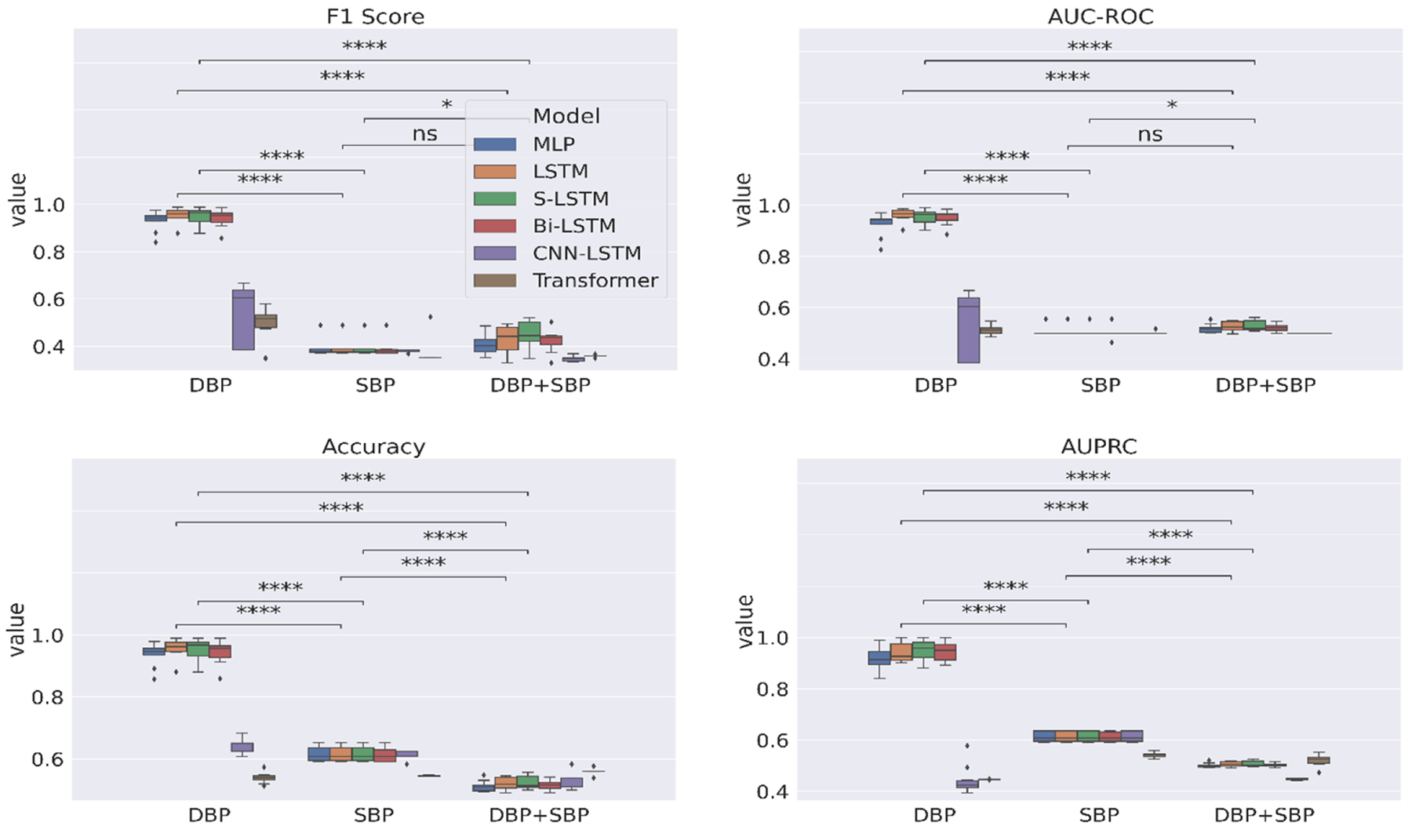
10-fold CV evaluation of deep learning models on the facet *feature*.

Lastly, Figure 6 compares the performance of deep learning models trained on three different features – DBP time series, SBP time series and multivariate time series with DBP and SBP observations (DBP+SBP). The DBP features alone are able to significantly improve the deep learning model’s generalization capability in most instances even without the incorporation of SBP observations.

## 5. Conclusion

A predictive model that can help with the identification of drug effect on patients has the scope to support clinical decisions that would enable more accurate prognosis and timely intervention for HF treatment. Towards this goal, in this work we propose annotation schemes that comprehensively incorporate medically relevant endpoints and biomarkers to precisely differentiate between cases of antihypertensive effectiveness and ineffectiveness across HF patients. We evaluate deep learning based predictive models on our annotated data which assert promising results and the utility of EHR for drug effectiveness studies.

We acknowledge that there are some limitations of this work that we would like to probe as future directions. In this study, we only use one data source in EHR (i.e., lab tests); while this eases the complexity of time series input modeling as lab measurements are inherently a continuous variable and also helps to directly embody information related to the patient’s physiological process conveniently, it falls short of capturing the heterogeneity in EHR as it fails to incorporate the phenotypic variables present in the other data modalities in EHR (e.g., notes). Integrating the physiological time series data with clinical notes and modeling jointly would open avenue to future investigations in drug effectiveness prediction.

## Figures and Tables

**Table 1.** Grid search considered values for hyperparameter- tuning.

Epochs	Batch size	Learning rate	Hidden dimension	Attention heads	Filters
[50–250]	[16, 32, 64]	[1e-03, 3e-03, 5e-05]	[10, 25, 50, 80]	[1, 5]	[10, 64, 100, 200]

## CCS CONCEPTS

- Computing methodologies → Artificial intelligence; Neural networks • Applied computing → Health informatics
